# Microbeam Radiotherapy—A Novel Therapeutic Approach to Overcome Radioresistance and Enhance Anti-Tumour Response in Melanoma

**DOI:** 10.3390/ijms22147755

**Published:** 2021-07-20

**Authors:** Verdiana Trappetti, Jennifer M. Fazzari, Cristian Fernandez-Palomo, Maximilian Scheidegger, Vladislav Volarevic, Olga A. Martin, Valentin G. Djonov

**Affiliations:** 1 Institute of Anatomy, University of Bern, 3012 Bern, Switzerland; verdiana.trappetti@ana.unibe.ch (V.T.); jennifer.fazzari@ana.unibe.ch (J.M.F.); cristian.fernandez@ana.unibe.ch (C.F.-P.); maximilian.scheidegger@ana.unibe.ch (M.S.); olga.martin@ana.unibe.ch (O.A.M.); 2 Department of Genetics, Department of Microbiology and Immunology, Faculty of Medical Sciences, University of Kragujevac, 34000 Kragujevac, Serbia; drvolarevic@yahoo.com; 3 Peter MacCallum Cancer Centre, Division of Radiation Oncology, Melbourne, VIC 3000, Australia; 4 University of Melbourne, Parkville, VIC 3010, Australia

**Keywords:** radiotherapy, melanoma, immune response, spatial fractionation, microbeam radiotherapy, synchrotron, oncology

## Abstract

Melanoma is the deadliest type of skin cancer, due to its invasiveness and limited treatment efficacy. The main therapy for primary melanoma and solitary organ metastases is wide excision. Adjuvant therapy, such as chemotherapy and targeted therapies are mainly used for disseminated disease. Radiotherapy (RT) is a powerful treatment option used in more than 50% of cancer patients, however, conventional RT alone is unable to eradicate melanoma. Its general radioresistance is attributed to overexpression of repair genes in combination with cascades of biochemical repair mechanisms. A novel sophisticated technique based on synchrotron-generated, spatially fractionated RT, called Microbeam Radiation Therapy (MRT), has been shown to overcome these treatment limitations by allowing increased dose delivery. With MRT, a collimator subdivides the homogeneous radiation field into an array of co-planar, high-dose microbeams that are tens of micrometres wide and spaced a few hundred micrometres apart. Different preclinical models demonstrated that MRT has the potential to completely ablate tumours, or significantly improve tumour control while dramatically reducing normal tissue toxicity. Here, we discuss the role of conventional RT-induced immunity and the potential for MRT to enhance local and systemic anti-tumour immune responses. Comparative gene expression analysis from preclinical tumour models indicated a specific gene signature for an ‘MRT-induced immune effect’. This focused review highlights the potential of MRT to overcome the inherent radioresistance of melanoma which could be further enhanced for future clinical use with combined treatment strategies, in particular, immunotherapy.

## 1. Introduction to Melanoma

Melanoma is a highly malignant type of skin cancer that develops from melanocytes, the cells that produce the UV-absorbing pigment melanin. Although melanoma constitutes only a minority of skin cancers, it is the deadliest [1]. During the last 50 years, the incidence of melanoma has dramatically increased and is now growing faster than any other cancer type. Worldwide, there are estimated to be around three new cases per 100,000 inhabitants per year; however, this picture varies by country and region [2]. The highest incidences are found in developed countries of the new world, such as Australia and New Zealand (around 35 new cases per 100,000 inhabitants yearly), but also in the US (approximately 30 new cases per 100,000 inhabitants were expected for 2020) [1]. Risk factors for melanoma include sun exposure, number of nevi, history of sunburns (especially during childhood), and light skin colour amongst other factors such as a genetic predisposition to photosensitivity [3].

Several subtypes of melanomas can be distinguished as mucosal, uveal, anorectal, meningeal, and the most common, cutaneous melanoma. Cutaneous findings such as superficial spreading, nodular, and lentigo maligna account for the vast majority of melanoma cases. The difference in prognosis between these subtypes is mainly determined by the stage of growth at the time of diagnosis [4]. Non-cutaneous melanomas are rarer and associated with a worse prognosis due to the delay in primary tumour discovery, high rate of relapse, and metastatic disease [5].

Most melanomas are detected clinically and primarily diagnosed by physical examination, dermoscopy, histopathology, and imaging, with the detection of circulating biomarkers being less crucial. Depending on the age of the patient and tumour thickness, sentinel lymph node biopsy is a powerful staging tool [6]. In addition, approximately 10 percent of all melanomas are initially diagnosed in the lymph node and are referred to as “unknown primary”. They are presumed to originate in the lymph node itself from pre-existing nodal nevi [7]. For all melanoma, early diagnosis is critical for improving prognosis as most complications with melanoma stem from metastases and their impact on the affected organ. Paraneoplastic syndromes may also occur, but at a much lower rate [8].

The severity of the disease is classified using four different stages according to the American Joint Committee on Cancer (AJCC) [9]. The staging system takes into consideration the thickness and ulceration status of the primary tumour as well as the presence of metastasis in the nearby lymph nodes and in distal sites. Stage I represents the lowest grade of severity defined by a small and localized malignancy, while stage IV is the highest grade of severity in which distant metastases are present and the clinical course is highly variable [10].

The main therapy for primary cutaneous melanoma is wide local excision. Depending on tumour thickness, a clinical margin of 0.5–2 cm is recommended for the excision in order to prevent local tumour recurrence [11]. To treat local or satellite metastases, surgical excision of the primary tumour and of metastases (in lymph nodes or distant organs) should always be performed whenever possible with curative intent. However, complete lymph node dissection is no longer recommended for patients with a positive sentinel lymph node in microscopic nodal disease, as there is no evidence that this improves prognosis. Nevertheless, in macroscopic nodal disease, complete lymph node dissection remains the key strategy [12,13]. Adjuvant therapy, such as chemotherapy and targeted therapies (e.g., inhibition of BRAF and c-KIT), are also employed for the treatment of melanoma. Melanoma has been shown to be an immunogenic tumour with polymorphic immune cell infiltration [14]. As a result, immunotherapies (e.g., IFN-α, interleukins) are also actively being explored, including novel immune checkpoint blockade and adoptive T cell therapy amongst others. Radiation therapy (RT) is another major therapeutic strategy for the treatment of many malignancies, however, it is often ineffective in the eradication of melanoma which is historically considered to be radioresistant. Here, we discuss the obstacles preventing efficient treatment of melanoma with RT, and how novel RT modalities, such as synchrotron generated, spatially fractionated microbeam radiation therapy (MRT) can overcome these obstacles. In particular, we discuss the role of conventional RT-induced immunity and the benefits of MRT’s stronger anti-tumour immune response. We present a comparative gene expression analysis from previously published studies of preclinical tumour models treated with MRT. The results of this analysis reveal a specific gene signature for an ‘MRT-induced immune effect’. Finally, we share our own experience about the successful treatment of mouse B16-F10 melanoma with MRT.

## 2. Melanoma and Radiation Therapy

### 2.1. Radioresistance of Melanoma and Conventional RT as a Treatment Strategy

The concept of melanoma radioresistance is based on early studies involving radiation cell-survival curves for rodent and human melanoma cell lines, which suggested an enhanced capacity to repair radiation-induced DNA damage [15,16]. Genetic analysis of 60 patients’ metastatic and non-metastatic melanoma samples revealed differential overexpression of genes involved in DNA double-strand break (DSB)/interstrand crosslink (ICL) repair, DNA replication, telomere maintenance, checkpoint activation, and also in nucleotide excision repair (NER) and base excision repair (BER) [17]. The overexpression of repair genes explains the resistance of melanoma to therapy. Another explanation was provided by Wu et al. [18], who analysed the response of the metabolome following ionizing radiation (IR) in mouse B16 melanoma cells. The levels of glutamate, alanine, glycine, and choline increased in irradiated cells. With a series of biochemical reactions upon the catalysis of these enzymes, more glycine can be synthesized which indirectly generates tetrahydrofolic acid, an important coenzyme in the DNA synthesis process. This finding supports that radiation tolerance of B16 cells is related to their efficient DNA damage repair.

The radiosensitivity of different melanoma cell lines varies significantly [19]. The radiation response in different tumours can be affected by multiple radiobiological parameters, such as hypoxic fraction, ability to deoxygenate, vascularization, cellular proliferation kinetics, number of tumour stem cells, and the inherent radiosensitivity of the tumour cells themselves [19,20]. Melanoma radioresistance has been found to positively correlate not only with efficient DNA damage repair, but also with a high fraction of hypoxic cells [21], long volume doubling time, slow growth, high cell loss, and low vascular density [22]. Matchuk et al. [23] attributed radioresistance of B16 melanoma to the efficient radiation response of a side population of tumour cells. In response to 3 Grays (Gy) of low-LET radiation, this population had less DNA DSBs compared to their differently located counterparts, were more quiescent, and had higher concentrations of nitric oxide (NO) which inhibits apoptosis.

Adjuvant RT has been shown to improve locoregional disease control in melanoma, but not overall survival, however, this still remains under investigation [24,25]. Currently, RT is used if surgery cannot be performed, or at the site of lymphadenectomy in node-positive melanoma patients [26]. Hypofractionation has been advocated as a promising treatment strategy. Early clinical investigations revealed that hypofractionation with higher doses (4–8 Gy per fraction) leads to better tumour control, compared to lower dose fractionation (2–3 Gy per fraction) [27]. Nevertheless, in the study of Lugade et al. [28], fractionated irradiation (3 Gy × 5) of mouse B16F10 tumours was only marginally effective compared to non-irradiated tumour-bearing controls, and a single dose of 15 Gy slowed tumour progression only at early stages after irradiation. Overall, advanced RT modalities, such as modern, hypofractionated radiation schedules, have not been shown to have an improved therapeutic impact. However, ablative RT modalities, such as stereotactic radiosurgery (SRS) and stereotactic body RT (SBRT) are commonly used in the management of in-transit or satellite metastases, preferably brain metastases [29]. Radiation is also used in palliative settings such as for treating spinal cord compression or painful bone metastases [29]. Complications from radiation in melanoma treatment, mainly fibrosis and oedema, seem to be clinically manageable. Most therapeutic guidelines suggest a case-by-case discussion of adjuvant treatment options after complete lymph node dissection [29].

### 2.2. Spatially Fractionated RT Including Synchrotron-Generated MRT

Technological advancements in RT have contributed to the improved efficacy of cancer treatment. These advancements are increasing the therapeutic ratio by successfully eradicating tumours while improving normal tissue sparing. Historically used to treat large, bulky tumours, spatially fractionated radiotherapy (SFRT) is a type of external beam radiation treatment that allows for the delivery of higher dose fractions of radiation in order to achieve local tumour control with minimal toxicity [30,31]. Clinically, SFRT was originally delivered using a homogenous beam of orthovoltage X-rays with a physical grid placed over the tumour resulting in non-homogeneous dose delivery [30,31]. This GRID therapy allowed for the delivery of higher doses while reducing damage to surrounding normal tissues, thus achieving superior outcomes in the palliative treatment of advanced-stage malignancies (Reviewed by Yan et al. [32]).

With advancements in technology, more sophisticated techniques were developed to deliver SFRT. GRID therapy could then be administered with megavoltage X-ray tubes where doses between 10 and 25 Gy were deliverable with minimal toxicity [32,33]. LATTICE RT is the evolution of 2D GRID therapy to a 3D configuration where high doses are delivered as independent vertices of incident radiation [34,35]. Distinct spatial configurations have also been developed to improve the therapeutic index of SFRT with millimeter and submillimeter fractionation. Minibeam RT delivers radiation as an array of parallel beams with widths of 0.5–0.7 cm spaced 1–3 mm apart using both proton [36,37] and X-ray [38,39,40] sources. Carbon nanotube technology is also being used to generate submillimetric beams with X-ray tubes [41].

Another type of SFRT that adopts submillimetre fractionation is MRT. Experimental synchrotron X-ray-generated MRT is delivered with the same dose deposition pattern of the minibeams, but using sub-millimetric beam widths of 25–100 μm spaced 200–400 μm apart, which drastically increases the normal tissue tolerance to radiation doses in the range of hundreds of Gy. This greatly exceeds the thresholds of conventional RT and the previously discussed SFRT regimens (reviewed by Fernandez-Palomo et al. [42] and Eling et al. [43]). In order to produce microbeams of this geometry, a beam with minimal divergence delivered at ultra-high dose rates is required, and therefore synchrotron sources are necessary [44]. X-ray microbeams of average 100 KeV beam energy (spectrum 50–600 keV) are delivered at dose rates of 10^4^ Gy/s up to 16 kGy/s [43,44]. Such dose rates trigger FLASH effects that confer additional biological benefits [45]. The biological FLASH effect was found to be reproducible when the whole dose of radiation is delivered in less than 200 milliseconds [46]. Synchrotron MRT is capable of generating the FLASH effect by delivering the highest peak doses compared to all other SFRT regimens within this time frame. Importantly, MRT promotes exceptional tumour control in conjunction with normal tissue sparing which has been well documented in a variety of models (reviewed in [42]). This includes melanoma, where remarkable tumour control was achieved in a murine model of B16F10 melanoma [47,48].

## 3. RT-Generated Immune Response

### 3.1. Conventional RT and Tumour Immune Response

Although IR is a type of sterile tissue injury, and IR-induced inflammation differs from the infectious inflammatory response, it is still a part of a basic immunological process; a protective response that aims to eliminate the cause of injury, remove damaged cells, and repair tissues. It is regulated by complex interactions among a variety of immune components and soluble mediators [49]. When tissues undergo radiation damage, the cells release a series of danger signals called damage-associated molecular patterns (DAMPs), which consist of fragments of cytosolic DNA, mitochondrial DNA, Calreticulin, ATP, and many others [50]. DAMPs commence a series of molecular cascades that lead to the activation of innate immune responses and immunogenic cell death [50]. Secretion of inflammatory mediators and cytokines is an initial, non-specific acute reaction, or “cytokine storm”, that usually resolves within 24 h [51]. Fractionated radiation creates a constant complex stress response, and a different cytokine profile [52]. The inflammatory innate immune response consists of monocytes, which accumulate at the damaged site and differentiate into dendritic cells (DCs), and inflammatory macrophages [53,54]. Macrophages support the local inflammatory process by a variety of functions such as phagocytosis of apoptotic bodies, presentation of antigens, cytotoxic activity and secretion of cytokines, reactive oxygen species (ROS), and NO [55]. NO affects vascular permeability, promotes tissue oedema, and is involved in inflammatory pain [56]. Later, cells of the adaptive immune system are activated, i.e., T-B-lymphocytes. T cells are responsible for cell-mediated immune responses, while B-cells play a role in the humoral immune response (mediated by antibodies). In contrast to the innate immune response, the adaptive immune response is characterized by high antigen specificity, latency, and the development of immunological memory [57].

It was long believed that the immune system did not contribute to the anti-cancer effects of IR. However, there is sufficient evidence showing that irradiated tumour cells become a robust source of antigen with adjuvant properties [54]. Through eliciting anti-tumour immune responses with diverse antigenic repertoires, IR is able to affect tumour progression, both locally and systemically. It is known that tumour control following RT is largely dependent on T cell responses [58]. Anti-tumour immune responses are triggered by the release of tumour antigens and pro-inflammatory factors that can promote DCs maturation and T cell activation. There have been reports of other mechanisms by which radiation increases immune responsiveness, including alteration in the secreted cytokine profile, expression of calreticulin (an “eat me” signal for cancer cells), and release of alarmins and nuclear proteins, including High mobility group box 1 protein (HMGB1) which serves as an endogenous ligand for toll-like receptor (TLR)-4 [59]. The activation of these signals leads to the death of cancer cells [60] and ultimately, strong anticancer immunity [61].

Conventional RT, however, is known to have a dual effect on the tumour immune response, where it can also elicit changes in the immune system that support tumour progression. Such changes may counterbalance the release of factors that stimulate the immune system to fight tumour cells. For example, radiation can enhance the release of factors that recruit immune-suppressing myeloid cells, including myeloid-derived suppressor cells (MDSCs), tumour-associated macrophages (TAMs), and tumour-associated neutrophils (TANs) [62]. RT also induces the release of colony-stimulating factor-1, CSF-1, from tumour cells, resulting in the expansion of TAMs and MDSCs. Irradiation of the primary tumour led to increased numbers of MDSC not only in the irradiated tumours, but also systemically, in peripheral blood, spleen, lymph nodes, and lungs [63]. This was driven by radiation-induced expression and secretion of CSF-1 that, when blocked with small molecule inhibitors of the CSF-1 receptor (CSF-1R), markedly reduced the expansion of myeloid cells and enhanced the response of the irradiated tumour to radiation. Radiation-induced activation of latent tumour growth factor (TGF)-β can trigger inhibitory mechanisms that can counterbalance the immune stimulatory effects of RT. TGF-β inhibits proliferation and effector function of CD8+ cytotoxic T lymphocytes (CTLs) and natural killer (NK) cells. Through suppression of the Jak-Stat signalling pathway, TGF-β induces G1 cell cycle arrest and down-regulates expression of NKG2D receptor, significantly reducing the cytotoxic potential of CD8+ CTLs and NK cells. Additionally, TGF-β may induce generation and expansion of MDSCs, promoting growth and metastasis of advanced tumours [64].

A review of Haikerwal et al. [54] comparing the immune effects of low- and high-dose radiation indicates that low-dose irradiation (less or equal to 2 Gy) induces DNA damage response (DDR), vascular normalization, and anti-inflammatory immune responses. The effects of low-dose radiation on the immune system may be subtle and may go unnoticed until long after exposure; these effects were comprehensively reviewed recently by Lumniczky et al. [65]. Delivery of high doses of radiation increases tumour antigen release, enhancing antigen presentation and T cell infiltration [58,66]. High-dose irradiation, such as a single dose of 5–10 Gy, or a hypo-fractionated (8 Gy × 3) RT schedule, induces cell death and pro-inflammatory responses that can support tumour clearance. Single high/ablative doses of RT (more or equal to 15 Gy) induce death via necrosis and/or senescence and are thought to be poor inducers of pro-immunogenic effects.

### 3.2. Combination of RT and Immunotherapy; Abscopal Effects

Activation of naïve T cells and their differentiation into effector T cells is regulated by the signals generated from the T cell receptor (TCR) complex and co-receptors (CD28, CD278, cytotoxic T lymphocyte-associated antigen 4 (CTLA-4) and program death 1 (PD-1)) [67]. CTLA-4 and PD-1 negatively regulate the effector function of activated T cells and PD-1 is mainly expressed on CD8+ CTLs where it is responsible for the alleviation of CD8+ CTL-dependent immune response upon elimination of viral antigens [67]. Additionally, activation of PD-1 in naïve CD4+ T cells induces their conversion to immunosuppressive CD4+ FoxP3+ T regulatory cells (Tregs) which, in a juxtracrine and paracrine manner, inhibit anti-tumour immune responses. PD-1 is, at least, partially responsible for Treg-dependent suppression of tumour-infiltrating CD8+ CTLs [67]. Accordingly, monoclonal antibodies which attenuate Treg-mediated suppression of CD8+ CTLs by causing inhibition of PD-1 signaling (Nivolumab and Pembrolizumab), have been used as new therapeutic agents in immunotherapy of solid tumours, including melanoma [68].

CTLA-4 is expressed on all subpopulations of activated T cells and has a dampening effect on both CD4+ and CD8+ T cell-driven immune responses [67]. CTLA-4 down-regulates expression of T-box transcriptional factors expressed in T cells (T-bet) and Eomesodermin (Eomes) which, consequently, inhibits the production of interleukin (IL)-2 and interferon (IFN)-gamma in CD4+ T cells and reduces the release of perforins and granzymes in CD8+ CTLs, significantly attenuating their pro-inflammatory and cytotoxic properties [69]. Since increased production of IL-2 and enhanced activation of T-bet and Eomes are crucially important for optimal activation and proliferation of CTLs, down-regulated expression of CTLA-4 promotes expansion and cytotoxicity of CTLs and has an important role in the enhancement of CTL-driven anti-tumour immunity [69].

In addition to activated CD4+ and CD8+ T cells, CTLA-4 is constitutively expressed on immunosuppressive FoxP3+ Tregs and plays a crucial role in Treg-driven suppression of anti-tumour immunity [67]. Tregs, in a CTLA-4-dependent manner, inhibit proliferation of CD4+ and CD8+ T cells and downregulate the expression of co-stimulatory molecules (CD80 and CD86) on DCs, attenuating their capacity for induction of effective anti-tumour immunity [67].

Although RT induces a generation of neo-antigens in irradiated tumours and, consequently, induces activation of neo-antigen-specific CD8+ CTLs, it also induces cell death in rapidly proliferating cells, including T lymphocytes [68]. Among T cells, FoxP3+ Tregs are the most resistant sub-population of T cells and their number is not strikingly different between irradiated and non-irradiated tumours. Accordingly, a decreased CD8+ CTL/Treg ratio is usually observed in irradiated tumours and is considered as one of the potential mechanisms for the detrimental effects of RT on anti-tumour immunity [70].

Since CTLA-4 and PD-1 negatively affect proliferation and cytotoxicity of CTLs and, at the same time, promote the generation and immunosuppressive properties of Tregs in irradiated tumours, the use of monoclonal antibodies, which induce a blockade of CTLA-4 (Ipilimumab) and PD-1 (Nivolumab and Pembrolizumab) opened a completely new avenue in cancer RT (reviewed by Demaria et al. [71] and Wang et al. [72]). Cellular make-up of solid and metastatic tumours (including melanoma) revealed a significantly elevated CD8+ CTL/Treg ratio followed by tumour growth delay and improved survival rate as a consequence of combined treatment with RT and Ipilimumab or RT and Nivolumab/Pembrolizumab [70,71].

The immune effects of the combined treatment show that the immune response is not only local but systemic. Thus, this idea could contribute to optimizing the combination of RT and immunotherapy for eradication of both the primary tumour and metastases via so-called abscopal effects. Abscopal effects are described as manifestations of systemic anti-tumour effects that lead to occasional cases of tumour regression outside the irradiation field, in which IR is used to immunize patients against their own tumours [73]. Strong interest in the mechanism underlying abscopal effects began when tumour regression was observed in melanoma patients undergoing palliative RT with, or immediately after, ipilimumab [74,75,76]. In a striking case, a patient with pulmonary metastatic melanoma treated with RT and ipilimumab showed a 30-fold increase in the titre of antibodies to metastatic melanoma (NY-ESO-1 protein) [76]. Further studies have associated abscopal effects with increased CD8+ T cells in peripheral blood [77] and also with a neutrophil-to-lymphocyte ratio of 2.6 or less [78]. Preclinical studies in breast and colon carcinomas showed that 3 fractions of 8 Gy and 5 fractions of 6 Gy on consecutive days (in combination with CTLA-4 blockade) were better than a single ablative dose of 20 Gy in inducing abscopal effects [74,79]. In a recent clinical trial, bulky tumours of the lung, kidney, skin, and prostate were partially irradiated with 1–3 fractions of 10–12 Gy of SBRT, without immunotherapy. This resulted in a curative effect: median shrinkage of the tumours (local bystander effect) was 70% in 96% of patients, whereas for the unirradiated metastases (abscopal effect), it was 50% in 52% of patients [80]. As an explanatory mechanism for this phenomenon, it was recently shown that more double-stranded DNA fragments accumulate in the cytoplasm of cancer cells with increasing radiation dose [79]. The presence of cytosolic DNA is detected by the cGas-STING pathway, which generates IFN-beta as an innate immune response that is normally associated with viral infections. Interestingly, abscopal effects are abrogated when irradiated cancer cells do not express cGAS-STING [79]. Moreover, increasing the radiation dose above 12 Gy also triggers the activation of Trex1, an endonuclease that degrades DNA DSBs preventing an immune response necessary to induce abscopal effects. However, repeated irradiation with doses that do not induce Trex1 can increase IFN-beta production, which recruits and activates Batf3-dependent DCs [79]. In addition, when the radiation dose was unable to induce Trex1 in several human and mouse cells, there was an increase in expression of IFN-inducible chemokines CXCL9, CXCL10, CXCL11, and CXCL16 [81]. This shows that the fine-tuning of the radiation treatment is crucial for the induction of abscopal effects.

### 3.3. Immune Implications of SFRT

The clinical benefits of SFRT have been well documented [32], but the radiobiological mechanisms governing improved treatment response have not been investigated in detail. Both clinical and preclinical data have implicated the immune system in the efficacy of SFRT, as observed by both tumour control and abscopal effects. Data from preclinical models support these observations of immune activation, showing increased secretion of inflammatory cytokines and elevated immune cell infiltration compared to un-irradiated tumours [66].

The key feature of SFRT in radiation-induced anti-tumour immunity is the peak-valley dose distribution, where the peaks are the regions of high-dose deposition and the valleys are the regions in between which receive relatively low doses [35,82]. This radiation scheme inherently results in the administration of ablative- and low-dose deposition simultaneously. The combination of a single, high ablative dose followed by subsequent lower doses with conventional RT resulted in the conversion of the immunosuppressive microenvironment to a more immunogenic one with increased infiltration of immune effector cells and a downregulation of regulatory T cells [83,84]. Detailed investigations differentiating the degree of inflammation and immune activation following SFRT vs. conventional methods have yet to be performed. It has been postulated that the high doses delivered in the incident or peak regions increase neo-antigen release which ultimately leads to T cell priming, while the low-dose regions in between, maintain circulation and perfusion to facilitate effector and T cell infiltration [82].

Given both inter- and intra-tumoral heterogeneity, effective doses vary between tumour types and within different tumour regions. Hypoxic regions will require higher ablative doses relative to oxygenated areas that are more susceptible to conventional-dose prescriptions [85]. Specific targeting of hypoxic regions using SBRT-PATHY was shown to trigger local and abscopal effects [80]. Recently, it has also been shown that SBRT can increase tumour response to PD-L1 inhibitor treatment [86].

### 3.4. Activation of the Immune Response Following MRT

#### 3.4.1. The Immune Response in Irradiated Tumours

Preclinical data from MRT studies have demonstrated intra-tumoural changes that provided a foundation for immune activation following this radiation modality. The activation of the local immune response playing an important role in the MRT-induced anti-tumour effect started to be clear since comprehensive transcriptomic studies of MRT-treated tumour models have been performed. So far, only a few studies, nonetheless collecting extensive biological data, have reported a comprehensive gene expression analysis: Sprung et al. [87], Bouchet et al. [88] (further analyzed in Bouchet et al. [89]), and Yang et al. [90]. These studies revealed that the biological processes associated with genes overexpressed after MRT irradiation, relative to un-irradiated controls, had a marked component clearly related to the modulation of the immune response.

At 24 and 48 h post-irradiation, several groups of genes and immunity-related-pathways were prominent in MRT-treated murine mammary tumours when compared to those treated with synchrotron broad beam (BB) radiation and un-irradiated controls [87]. Importantly, within the pathways related to MRT, relative to BB, upregulation of genes related to inflammation, IFN signalling, immune response-antigen presentation and IFN-gamma signalling were statistically significant. On the other hand, MRT was also shown to downregulate some immunity-related genes compared to BB irradiation, such as those in the major histocompatibility complex (MHC) class II antigen gene family members, which are associated with antigen presentation to CD4+ T-helper cells. In a study performed on the cell line EMT6.5, the same that was implanted in the mouse mammary gland, Yang et al. demonstrated that at 24 h post-irradiation gene expression profile and molecular pathways in MRT-treated cells were significantly different from those treated with conventional RT (irradiation up to 7.5 Gy with a Cobalt-60 teletherapy unit) [90]. Interestingly, the authors also reported that MRT treatment at a high peak dose (560 Gy) induced different radiation responses relative to lower doses (112 or 280 Gy). Moreover, MRT was found to upregulate the pathways involved in inflammation and lymphocyte activation [90].

Similarly, early transcriptional changes following MRT of rat glioma revealed an increase in genes associated with inflammation, including those associated with NK or CD8+ lymphocytes, in irradiated tumours and surrounding normal tissues [88]. Subsequently, in the same model, differential gene expression following MRT has been shown to induce the overexpression of transcripts indicative of the presence of DCs in the tumour relative to the normal tissue, from hours and up to 15 days post-irradiation, implicating subsequent activation of the adaptive immune system following MRT [89]. Surface markers of monocytes and macrophages were also represented in differential gene expression in the tumour tissue following MRT, further suggesting immune cell recruitment to tumour after irradiation [89].

Prior to the genomic studies, evidence that the immune system may be involved in the MRT therapeutic effect came from a study reporting that gene-mediated immunotherapy enhances the efficacy of MRT in terms of survival for rat gliosarcoma [91]. One of the mechanisms that stand behind the enhanced infiltration of immune cells after MRT treatment is the disruption of tumour vasculature. It has been shown in the zebrafish model that neutrophils adhere to the endothelium of blood vessels in correspondence to the rupture caused by the microbeam path [92]. This process was documented at early time points (6–48 h post-irradiation), and immature vasculature was found to be more susceptible to the MRT vascular-disruptive effect. Tumour vasculature, being immature due to rapid and dysregulated tumour growth, was selectively destroyed by MRT in murine melanoma [47]. Five days post-irradiation, blood vessel perfusion was lower in MRT compared to BB and un-irradiated controls, indicative of the presence of an earlier mechanism of vascular disruption [47]. A decrease in vessel density after MRT was also observed in a murine mammary tumour model [93], while in rat gliosarcoma an increase of blood vessel permeability at 5 days post-MRT was documented and followed by a decrease in tumoral blood volume fraction 3 days later [94]. The rupture of the vasculature may facilitate the infiltration of several types of immune cells, not only neutrophils as observed in zebrafish, to the tumour microenvironment at different times before inducing the irreparable blockage of the vessels. For this process to happen, high peak doses (400 Gy and over) are required. On the other hand, when lower peak doses of 100–150 Gy were applied, MRT did not permanently disrupt the vasculature but created a transient vascular permeability window, that can be exploited to deliver chemotherapeutic drugs and/or nanoparticles to the tumour site [95]. This will contribute to the amelioration of tumour control when a second therapeutic MRT fraction with a higher peak dose (400 Gy peak) is applied [95].

Important work contributing to the understanding of MRT-induced immune responses came from the Rogers group in 2019 [96]. In this study, murine EMT6.5 mammary tumours were MRT-irradiated, and the local immune response was evaluated 48 h post-irradiation in comparison to the BB treatment control. After MRT, there was a decrease in TAMs and TANs [96]. On the other hand, the frequency of T cell infiltration into the tumour microenvironment increased. This has also been observed in a mouse melanoma model [47]. This suggests that MRT is able to reduce the pro-tumourigenic response while increasing the anti-tumourigenic response after radiation. This is in contrast to conventional RT, where a dual response eliciting both anti-tumourigenic and pro-tumourigenic immune responses is observed. Another interesting result coming from this research is the increased expression of mRNA levels of the *CD11c* transcript, which might be indicative of a higher infiltration of DCs into the tumour post-MRT, relative to the BB control. Lastly, the mRNA expression level for *Arg1*, a classic marker for a subset of M2–like immunosuppressive TAMs [97], is higher after high peak dose MRT treatment (560 Gy). In a whole-genome study performed by the same group [87] in the same mammary tumour model, it was also observed that at 24 and 48 h post-MRT in comparison with BB treatment, expression of *Mrc1* and *CD163,* markers for M2-like macrophages [98], also increased. It is highly unlikely though, that recruited macrophages at the tumour site after radiation injury could differentiate towards an M2-like phenotype in only 48 h. Mrc1, alias CD206, is expressed by phagocytic macrophages [99], as well as CD163 [100]. In the light of this result, we hypothesize that it might be plausible that tumour-associated macrophages already present at the moment of the irradiation, were driven towards a more phagocytic phenotype by MRT and might contribute to the clearance of apoptotic cells. Additionally, in murine melanoma [47] and in rat glioblastoma [101] frequencies of CD68+ macrophages increased after MRT treatment compared to BB. However, phenotypic differentiation of macrophages was not investigated.

Finally, it is not clear yet if the suppression of pro-tumourigenic immune responses elicited distinctly by MRT in the murine EMT6.5 model is reproduced in all other types of cancer when irradiated with MRT, but it is obvious that there is a better anti-tumourigenic response (increase of T cells) relative to BB treatment. The shifted balance towards a more anti-tumourigenic and pro-inflammatory phenotype would explain the better tumour control observed so far in response to MRT treatment. Future studies are required to give a detailed phenotypic characterization of all the immune subpopulations that play a role in the tumour, including B cells, DCs, etc., with an emphasis on the temporal dynamics.

#### 3.4.2. The Immune Response in Normal Tissues

The immune response after MRT has also been investigated in normal tissues. Leukocyte infiltration was observed to be higher after BB than after MRT irradiation in normal mouse skin at 5 days after irradiation [102]. On the other hand, general infiltration of leucocytes was found to be higher after MRT in the dermis of the mouse ear pinnae following MRT when compared to un-irradiated controls [103]. This leucocyte infiltration was composed of neutrophils, lymphocytes, and monocytes [103].

In rat brain tissues, 6 h after MRT irradiation, the following genes (selected among the top ten in terms of differential fold-change) were overexpressed compared to un-irradiated controls: *Ccl2*, *Cxcl10* (Interferon gamma-induced protein 10), *Cxcl11* (interferon-inducible T-cell alpha chemoattractant), *Gbp2* (interferon-induced guanylate-binding protein 2), *Ccl9* (macrophage inflammatory protein-1 gamma, MIP-1γ), *Sele* (encoding E-selectin, also known as endothelial leukocyte adhesion molecule 1), and *Ccl7* (monocyte-chemotactic protein 3) [88]. All the genes are IFN inflammatory response-related and/or promote leukocytes attraction and infiltration. Furthermore, the transcript for the cytokine Ccl2 was the most upregulated with a staggering fold increase of 185.7 times with respect to the un-irradiated brain tissue [88].

In a non-tumour setting, MRT irradiation has been shown to generate systemic effects in mice [104,105,106]. When only a fraction of a mouse hind leg was irradiated, DNA damage, apoptosis, local inflammation, and senescence were elevated and cellular proliferation decreased in out-of-field normal tissues [104]. These effects were accompanied by the innate immune response at the irradiated skin patch and by both innate and adoptive immune responses at the distant sites. Gene expression analysis of out-of-field skin samples confirmed that genes associated with inflammation and DNA damage were involved in the propagation of these systemic effects [106]. The functional immune system seems to be necessary for such effects to occur since little or no changes in DNA damage and apoptosis were found in out-of-field tissues from immune-deficient SCID/IL2γR^−/−^ (NOD SCID gamma) mice and mice with disrupted macrophage recruitment (CCL2 KO and injected with antibodies to CSF-1R), compared to their immunocompetent counterparts [105]. The hypothesis that the innate immune response is specifically required to initiate abscopal effects has been confirmed by a study in which athymic mice irradiated with MRT showed an abscopal effect despite the absence of adaptive immunity [107].

## 4. Comparative Gene Expression Analysis in MRT-Irradiated Tumours

**Figure 1:**
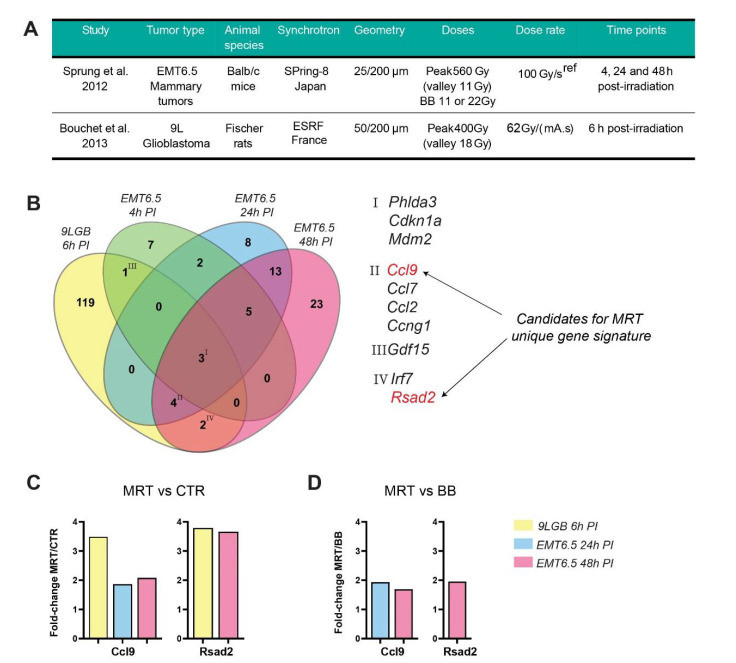
Comparative gene expression analysis in MRT-irradiated tumours. (**A**) Table reporting the most important experimental parameters of the two compared studies (Sprung et al. [87] and Bouchet et al. [88]). ‘*ref*’ in (**A**) is reference [118]. (**B**) A Venn diagram displaying the common genes that are overexpressed after MRT in the two studies. For both studies, genes indicated as significantly modified were selected based on a fold-change ≥ 1.8 compared to un-irradiated controls. For the 9L glioblastoma (GB) study, gene expression was analyzed at 6 h post-irradiation (PI), while for the EMT6.5 mammary tumour study, three different time points were considered, 4, 24, and 48 h PI. These three different time points are reported as separate data sets in the diagram along with the single glioblastoma gene dataset. Common genes among the different groups are underlined in four different clusters, I to IV, and reported on the right side of the panel. In red are reported genes *Ccl9* and *Rsad2*, which were significantly overexpressed in the comparison of MRT vs. BB analyzed in the EMT6.5 study. The VennPainter software was used to create the Venn diagram [109]. (**C**) The gene expression fold-change in tumours after MRT over the unirradiated control for two underlined genes *Ccl9* and *Rsad2*. (**D**) The fold-change of the comparison MRT vs. BB from the study on EMT6.5 tumours for genes *Ccl9* and *Rsad2*.

Only two different in vivo tumour models were employed for comprehensive transcriptomic analysis within the above-mentioned studies. Specifically, Sprung et al. [87], used a mouse model of mammary EMT6.5 tumours inoculated subcutaneously in the right hind leg of Balb/c mice. The study of Bouchet et al. [88] employed an orthotopic 9L glioblastoma model in Fischer male rats. For the first time, we performed a comparative gene expression analysis between these two studies, aiming to find a specific MRT gene expression signature. Experimental parameters used in the two selected studies are shown in Figure 1A. Illumina BeadChip was used for the whole genome analysis of the mammary tumours [87], and an oligonucleotide microarray for detecting 28,000 genes with the Affymetrix GeneChip Scanner 3000 was used in the rat glioblastoma study [88]. Normalized gene expression data from both studies were analyzed in search of similarities. Data were obtained from the available online supporting information of the two publications: Tables S2A–C, S3A–C and S4A–C from [87] and Table S1 from [88]. For the latter, genes indicated in the publication as significantly modified and with an increased fold-change post-MRT ≥ 1.8 were selected. Rat gene symbols were converted to mouse gene symbols using the BiomaRt package in R [108] and for four genes, a correspondence in mouse was not found. The same fold-change selection was applied for the EMT6.5 study and genes were also selected with a *p*-value < 0.05. Following this filtration, gene lists coming from the group 9L glioblastoma evaluated at 6 h post-irradiation (9LGB 6 h PI) and from the three groups of EMT6.5 mammary tumours investigated at 4-, 24- and 48-h post-irradiation (EMT6.5 4 h PI, EMT6.5 24 h PI and EMT6.5 48 h PI respectively) were intersected in a Venn diagram (Figure 1B) using the software VennPainter [109]. Four different clusters of common genes between the different selected gene lists were underlined (Figure 1D right panel).

In Cluster I, we found three genes that are significantly overexpressed after MRT relative to control in all the four groups analyzed. These genes are *Cdkn1a*, *Mdm2*, *Phlda3* and they are all known to be upregulated in response to radiation [110]. In Cluster II, we identified four genes: *Ccl2*, *Ccl7*, *Ccl9,* and *Ccng1* that are common to the groups 9LGB at 6 h, EMT6.5 at 24 h, and EMT6.5 at 48 h post-irradiation. The first three genes are involved in myeloid cell recruitment [111,112], while *Ccng1* is involved in cell cycle arrest in response to DNA damage [113]. Cluster III includes only the gene *Gdf15*, common to the groups 9LGB at 6 h and EMT6.5 at 4 h post-irradiation. Growth/differentiation factor-15 (GDF-15), also known as macrophage inhibitory cytokine-1 has been recently reviewed as a possible target for cancer immunotherapy [114]. In Cluster IV (intersection between 9LGB at 6 h and EMT6.5 at 48 h post-irradiation), we underlined the interferon-dependent genes *Irf7* and *Rsda2*.

The comparison only assessed MRT vs. un-irradiated tumours, since in the study by Bouchet and colleagues [88] no treatment control was included. However, we wanted to know whether some of the genes belonging to the four clusters would have a specific response to MRT and not to BB irradiation. In order to do this, we cross-checked our set of 10 genes with the genes that were found specifically overexpressed for the comparison of MRT vs. BB reported in the study by Sprung and colleagues in the mammary model [87]. We found a match for two genes specifically overexpressed after MRT when comparing them to the BB treatment control. These two genes are *Ccl9* and *Rsad2* (their fold-change measured in the two studies is reported in Figure 1C,D). The latter is an IFN-inducible antiviral protein triggered by type-I and type-II IFN [115]. It has been demonstrated by Vanpouille-Box and colleagues that the ability of RT to induce abscopal effects is strictly related to the activation of the type-I IFN pathway [79]. Indeed, they found in mammary adenocarcinomas that *Rsad2* was upregulated 24 h after the RT regimen that showed induction of abscopal effects (8 Gy × 3). In the same settings, *Ccl2* and *Ccl7*, found in our Cluster II, and *Irf7* in our Cluster IV together with *Rsda2*, were upregulated [79].

CCL9 is a chemokine that induces recruitment of myeloid progenitor cells at tumour sites and it has been detected as a possible target for treating metastatic disease [116]. Remarkably, the cytokine CCL9, alias MIP-1 (macrophage inflammatory protein-1 gamma), was found significantly overexpressed also in the microenvironment of mouse melanomas at 5 and 9 days post-irradiation when compared to the expression in BB treated or untreated tumours [47] and it is among the top ten overexpressed genes in normal brain tissue after MRT [88]. In addition, *Ccl9* mRNA expression was validated by qPCR in Ibahim et al. [117], and it was confirmed to be higher in MRT with 560 Gy peak dose than in the other treatments and un-irradiated controls. These additional pieces of data corroborate our findings.

If we consider that we examined two different animal species (rat vs. mouse) and two different tumour types (glioblastoma vs. mammary carcinoma) located in different organs (brain vs. skin), the similarities are quite remarkable. Certainly, what stands out is the similarities between the early time point of 6 h post-irradiation in the glioblastoma model and the later time points of 24 and 48 h in the EMT6.5 model. It seems that in rats the transcriptional changes induced by MRT happen more quickly than in mice.

## 5. MRT as a Novel Strategy for Treatment of Melanoma

A recent research interest of our group is exploring the efficacy of MRT for treating melanoma. An ad hoc preclinical B16F10 radioresistant melanoma model for testing a microbeam array was established in our laboratory [119]. In the very first study, it was clear that MRT elicits an extraordinary delay of tumour growth when compared to a BB irradiation or to un-irradiated controls [47]. Aside from the underlined mechanisms of vascular disruption and induction of cellular senescence, we demonstrated that MRT elicits superior tumour control due to the induction of a potent tumour immune response. Especially, MRT induces a significant increase of monocyte-attracting cytokines (MCP-1, MIP-1a, MIP-1b, RANTES), and IL-12p40 in the melanoma microenvironment [47]. MRT also promoted infiltration of macrophages, CD4+ and CD8+ T cells and NK cells, both in the periphery and within the irradiated tumour 5–12 days after treatment. This was observed in contrast to naïve or BB-irradiated tumours [47].

In another study, Fernandez-Palomo and colleagues compared the efficacy of delivering three fractions of 133 Gy MRT, administered in three consecutive days versus only one MRT fraction of 400 Gy in a single day [48]. Remarkably, after the temporally fractionated irradiation, 50% of melanomas underwent complete tumour remission. For an 18 months period, the mice showed no sign of tumour re-growth, and after they were sacrificed, immunohistochemical analysis of the tumoural vestige revealed a complete absence of melanoma cells and the presence of melanophages. Melanophages were described as very large melanin-laden cells, positive for macrophage markers [48].

The importance of this result is not only that radioresistant B16F10 melanoma does not lose sensitivity to the radiation treatment after several fractions, but also that there is no local tumour recurrence and metastasis for a long period of time. This could be explained by the presence of the anti-tumour abscopal effect specifically triggered by fractionated MRT. Therefore, this work offers a unique model to further study MRT-induced abscopal effects and is a starting point for optimizing a treatment protocol that could increase the rate of tumour remission above 50% in this preclinical model.

**Figure 2:**
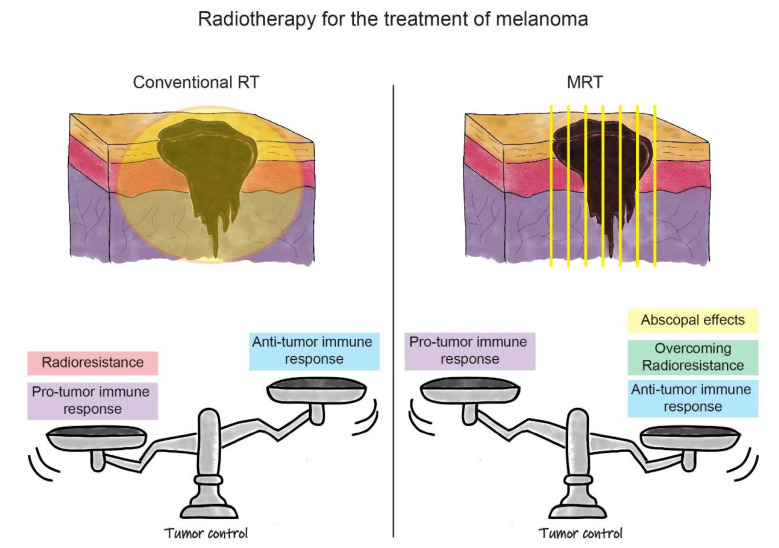
Schematic representation of the RT strategies for the treatment of melanoma. In the upper part of the figure, there is a schematic representation of melanoma irradiation with conventional RT (on the **left**) and MRT (on the **right**). In the lower part, the balance between positive and negative effects influencing treatment outcomes are represented (i.e., tumour control) for both RT modalities. On the left, we can see how the pro-tumour immune response, associated with radioresistance, outbalances the anti-tumour immune response induced by conventional RT. This leads to the current uncommon use of conventional RT for the treatment of melanoma. In the lower right panel, it is shown how the positive effects of MRT, such as induction of the anti-tumour immune response, overcoming melanoma radioresistance, and possible triggering of abscopal effects, shift the balance towards a better outcome for melanoma eradication. This promotes MRT as a new opportunity for melanoma treatment in future clinical settings.

In Table 1, we summarize findings in the field of MRT-induced tumour immune responses in melanoma and other cancers that were reported in this review. In Figure 2, we outline the advantages and disadvantages of conventional RT and MRT for the treatment of melanoma. In the case of MRT, the factors discussed above, such as the induction of the anti-tumour immune response, overcoming tumour radioresistance, and the ability to generate the anti-tumour abscopal effects, contribute to the treatment success in melanoma. We have yet to optimize the fractionation schedule on the basis of normal tissue tolerance and tumour response. In parallel, the success of MRT in melanoma treatment can be further boosted by exploiting the role of IR in the modulation of local and systemic immune processes. The treatment improvement will be based on enhancing the anti-tumour immune response in combination with MRT. This can be done directly by targeting immune checkpoints, or indirectly, by blocking the upstream of the innate (‘frontline’) immune response. The proof of concept of these treatment combinations will open a completely novel avenue for the treatment of radioresistant tumours beyond melanoma.

## 6. Conclusions

Despite the progress made in the last decades, the efficacy of RT remains suboptimal for many radioresistant tumours and invariably leads to a host of normal tissue complications. MRT is an innovative strategy that could shift the current RT practices into a new era. The therapy has the potential to significantly improve tumour control or even result in complete ablation, while preserving the normal/healthy tissues, even after receiving MRT peak doses of thousands of Gy. MRT, therefore, has the potential to overcome radioresistance, modulate the local immune response and trigger abscopal effects. These unique features prompt us to propose MRT as a novel radiotherapeutic approach with the potential to treat inoperable radioresistant lesions. MRT veterinary trials should be the first step taken towards clinical translation. Thereafter, the use of MRT as a primary treatment strategy or as a neoadjuvant in combination with systemic therapies, in particular, immunotherapies, should be explored in extensive clinical trials. In an ideal future scenario, MRT would reduce the size of the primary tumour and prevent organ metastases by triggering the systemic anti-tumour response before surgical removal.

## Figures and Tables

**Table 1:** The most important findings in the field of MRT induced response in different tumour models.

**Tumor Model**	**Assay Type**	**MRT Effects on Tumor Immune Response**	**Reference**
Glioblastoma in rat	Oligonucleotide microarray	Upregulation of genes associated with inflammation, NK or CD8+ T cells	Bouchet et al., 2013 [88]
Glioblastoma in rat	Oligonucleotide microarray	Upregulation of transcripts indicating the presence of DCs, monocytes, and macrophages	Bouchet et al., 2014 [89]
Glioblastoma in rat	IHC	Increase of infiltrated macrophages	Eling et al., 2021 [101]
Mammary EMT6.5 in mouse	Whole genome analysis	Upregulation of genes related to inflammation, IFN signalling, antigen presentation	Sprung et al., 2012 [87]
Mammary EMT6.5 cell line	Whole genome analysis	Upregulation of pathways involved in inflammation and lymphocyte activation	Yang et al., 2014 [90]
Mammary EMT6.5 in mouse	Flow cytometry, IHC	Decrease in tumour-associated macrophages and neutrophils; increase of infiltrated T cells	Yang et al., 2019 [96]
Melanoma B16F10 in mouse	Cytokine BioPlex analysis, IHC	Increase of monocyte-attracting cytokines; Increase of infiltrated macrophages, CD4+ and CD8+ T cells, NK cells	Potez et al., 2019 [47]
Melanoma B16F10 in mouse	IHC	Presence of melanophages at the place of tumor cells and absence of metastasis up to 18 months post-treatment	Fernandez-Palomo et al., 2020 [48]

## Data Availability

The data presented in this study are available on request from the corresponding author.
